# Maximizing the Inner Resilience of a Network-on-Chip through Router Controllers Design †

**DOI:** 10.3390/s19245416

**Published:** 2019-12-09

**Authors:** Douglas R. Melo, Cesar A. Zeferino, Luigi Dilillo, Eduardo A. Bezerra

**Affiliations:** 1 Laboratory of Embedded and Distributed Systems (LEDS), University of Vale do Itajaí, Itajaí 88302-902, Brazil; 2 Space Systems Research Laboratory (SpaceLab), Federal University of Santa Catarina, Florianópolis 88040-900, Brazil; 3 Laboratoire d’Informatique, de Robotique et de Microélectronique de Montpellier (LIRMM), University of Montpellier, CNRS, 34095 Montpellier, France

**Keywords:** systems-on-chip, networks-on-chip, router architecture, fault tolerance

## Abstract

Reducing component size and increasing the operating frequency of integrated circuits makes the Systems-on-Chip (SoCs) more susceptible to faults. Faults can cause errors, and errors can be propagated and lead to a system failure. SoCs employing many cores rely on a Network-on-Chip (NoC) as the interconnect architecture. In this context, this study explores alternatives to implement the flow regulation, routing, and arbitration controllers of an NoC router aiming at minimizing error propagation. For this purpose, a router with Finite-State Machine (FSM)-based controllers was developed targeting low use of logical resources and design flexibility for implementation in FPGA devices. We elaborated and compared the synthesis and simulation results of architectures that vary their controllers on Moore and Mealy FSMs, as well as the Triple Modular Redundancy (TMR) hardening application. Experimental results showed that the routing controller was the most critical one and that migrating a Moore to a Mealy controller offered a lower error propagation rate and higher performance than the application of TMR. We intended to use the proposed router architecture to integrate cores in a fault-tolerant NoC-based system for data processing in harsh environments, such as in space applications.

## 1. Introduction

Due to technological development and increasing integration, communication architectures that are used in computers for aerospace applications are composed of a growing number of processing cores. The increase in processing power is a demand due to the increasing amount of high resolution sensors and the bandwidth requirements of satellite-ground links. Some approaches proposed in the literature do not fulfill the communication requirements of future on-board computers [1]. Network-on-Chip (NoCs) represent an alternative to the interconnect bus for multi-core systems. They can be used in aerospace applications as the communication backbone for interconnecting processors, memories, and the controllers of actuators and smart sensors when these components are integrated on a single chip to reduce the dimensions of the primary computer systems. However, the costs of an NoC are not negligible, especially when fault tolerance is required.

According to [2], different types of System-on-Chip (SoCs) require fault-tolerant components depending on the target environment. A fault-tolerant NoC must be able to detect the occurrence of a fault and prevent the resulting error from causing an application failure. However, providing reliability in an NoC affects performance, silicon costs, and power consumption, as this is usually done through redundancy.

Currently, fault tolerance in NoCs mainly relies on spatial redundancy and data encoding. However, as replication increases power dissipation, in energy constrained systems such as embedded and aerospace applications, it is necessary to look for solutions that allow fault tolerance with low energy impact [3].

In this context, this work aims at evaluating the performance and resilience of an NoC router using combinations of flow regulation (or flow control), routing, and arbitration controllers, presenting the possible trade-off between the use of hardware resources and the susceptibility to error propagation. The results showed that the use of Mealy Finite-State Machine (FSMs) to implement the controllers provides a significant reduction in the number of propagated errors, at the price of reducing the maximum operating frequency and increasing the energy consumption of the router.

The main contribution of this work does not concern the application of specific hardening techniques for a given architecture, but rather to assess the impact of different implementations on the inner resilience capacity of the router itself in terms of error propagation. Since the focus of the work is on the controllers, other sequential logic structures (e.g., buffers) have remained unchanged across all architecture combinations. As far as we know, this is the first study investigating the design of internal controllers to improve the reliability of NoC routers.

The remainder of this paper is structured as follows. Section 2 provides a description of NoCs characteristics and fault tolerance. In Section 3, we present the router architecture developed for this evaluation. Section 4 presents the verification model and the fault injection campaign used for simulation. Finally, Section 5 discusses the experimental results, and Section 6 summarizes the conclusions of the article.

## 2. Background

**Figure 1:**
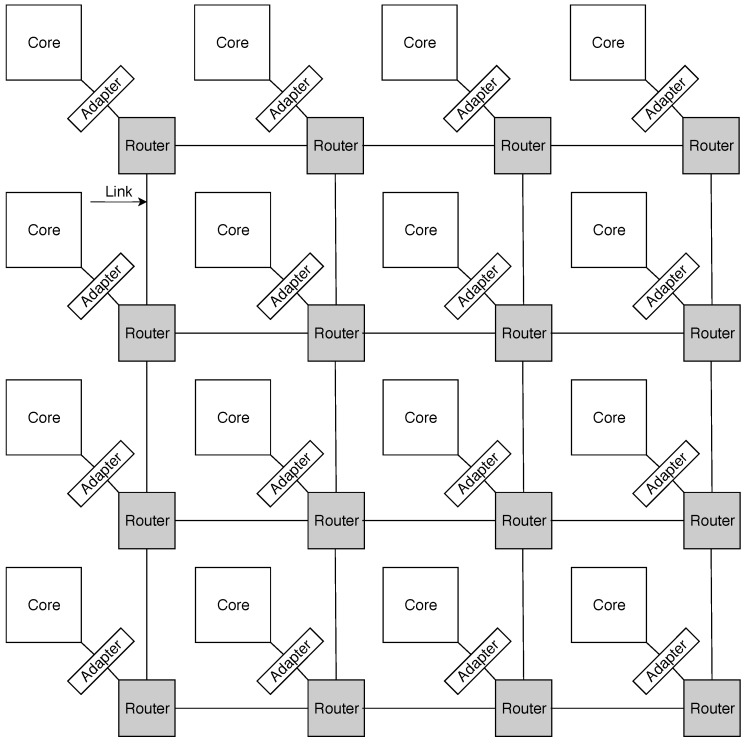
A 4 × 4 NoC-based system [11] (©2019 IEEE).

The interconnection in an SoC with few cores is generally performed through shared buses because this architecture is reusable and reduces design costs and time. However, SoCs with dozens of cores require an interconnection structure with performance scaling adjusted to the size of the system. For this reason, in the early 2000s, several studies argued that NoCs would be the best means of solving this problem [4,5,6,7,8]. NoCs are derived from the interconnection networks used in parallel computers [9,10]; they are reusable, as the shared bus, and offer parallelism in communication and scalable performance. An NoC consists of routers, links, and adapters (or Network Interfaces) [8], as shown in Figure 1.

The router is the main component of an NoC system. It comprises registers, multiplexers, arbiters, routing, and flow control circuits, in addition to buffers for the temporary storage of packets [10]. It also has input and output links for communication with the local core and other routers in its neighborhood. The links are usually structured into two unidirectional point-to-point channels, which may be synchronous or asynchronous. Each channel is made up of data and flow control signals [8].

The Network Interface (NI) is the unit that transparently connects a processing core to a router. It is responsible for adapting the communication protocols used by the core and the NoC. NIs are classified following the nature of the client, which might be a processor, a shared memory, or an external channel [10].

### 2.1. Network-on-Chip Communication

The usual means of communication between cores in an SoC is through an exchange of messages broken into packets. Each packet consists of a header (relative to the start of the packet), a payload (content), and a trailer (signaling the end of the packet).

The data flow in the network is outlined in [12]. Table 1 identifies the components of an SoC and relates to the layers of the Open System Interconnection (OSI) model. Each layer can provide services, including fault tolerance. The System layer corresponds to the processing cores and the application. The Interface layer decouples the cores from the network and controls the process of sending and receiving packets. The Network layer is responsible for packet routing, while the Link layer addresses questions related to coding, synchronization, and reliability.

### 2.2. Network-on-Chip Features

An NoC can be characterized in terms of the following attributes [8]: topology, flow regulation, memorization, routing, arbitration, and switching.

The topology defines the routers and links and arranges them in the form of a graph. The most common topologies in SoCs are those of the planar type, like the 2D mesh. With the advent of 3D integration processes, NoCs for these systems usually have inter-layer communication through vertical links. These links are made available in reduced numbers and are known as Through-Silicon Via (TSV) [13]. The topology of an NoC defines the physical layout and the connections between the nodes and the channels. It is characterized by the number of ports in each router, the number of hops from the source node to the destination node, the channel bandwidth, and path diversity [14]. The architecture of an NoC is usually defined at design time or according to the requirements of the target application [15].

The flow control is responsible for the allocation of the resources necessary for a packet to travel through the network, by regulating traffic in the channels. This regulation is required to prevent any undesired loss of data from a sender to a receiver. In general, NoCs are networks that do not discard packets (i.e., they are lossless). Packets are generally divided into flow control unit (flits), on which the flow control takes place. Different flow control techniques are employed in NoCs, such as handshake, stop-and-go, credit based, and virtual channels. Typically, a flit corresponds to a word of the physical channel, called physical unit (phit).

Packets destined for channels that are already allocated must wait before being forwarded. This approach requires the implementation of a scheme that enables the blocked packets to be stored in queues within the router. Memorization (or buffering) can either be implemented at the input channels or the output channels. A shared centralized memory based approach can also be adopted.

The routing sets out the path that must be followed to forward a packet to the destination. There are many different routing strategies, which are usually classified in accordance with the following criteria: the number of destinations (unicast or multicast), the location where the routing is performed (centralized, source, or distributed), the type of physical implementation (table based or an algorithm), and its adaptiveness (deterministic, adaptive, or oblivious). An example of a widely used technique in NoCs is dimension order routing, which is characterized as unicast, deterministic, and usually implemented as a hardware supported algorithm executed in the routers. Routing algorithms in NoCs should prevent packets from being blocked in the network (causing a deadlock) and the problem of packets moving through the network without reaching their destination (livelock) [9].

A conflict that arises when two or more packets compete for the same channel can be settled by arbitration. Round-robin is the arbitration scheme that is most widely employed in NoCs because it provides a fair distribution of channel usage. In the case of NoCs with Quality of Service (QoS) provision, requiring flow differentiation, alternative schemes can be used to meet the temporal requirements.

Switching determines how a message is transferred from the input of a router to one of its output channels. The main types are circuit and packet switching. There are different types of packet switching techniques, such as Store-and-Forward (SAF), Virtual Cut-Through (VCT), and wormhole. The latter performs the switching in the flit-level, and it is the most commonly used in NoCs because it offers low latency at less cost.

### 2.3. Fault Tolerance and Networks-on-Chip

The relationship between fault, error, and failure was presented in [16]. A fault may manifest an error, and an error may result in incorrect behavior, called a failure. Faults and errors can be masked and not lead to an error or failure. Masking occurs on logical, architecture, or application levels and for a variety of reasons. Faults and errors can be classified according to their duration and can be transient, intermittent, or permanent [17].

Studies about fault tolerance in NoCs mainly address both transient and permanent faults. For instance, the works in [18,19,20,21,22,23] examined Single Event Upset (SEU) in NoC components. The studies in [18,19,20] dealt with transient faults that were due to *crosstalk*. In [24,25,26], the authors investigated the problem of short and open circuit faults in the links of an NoC. In [27,28,29], the authors discussed the yield of vertical links in 3D NoCs. The studies that examined intermittent faults [24,30,31] treated them as permanent faults.

Following, we discuss the way fault tolerance techniques are deployed in NoCs, the methods for the detection, correction, and recovery of errors, diagnosis, and repair, and the metrics commonly employed to evaluate these techniques and methods.

#### 2.3.1. Network-on-Chip Layers

An NoC can be divided into four layers: System, Interface, Network, and Link (Table 1). The studies examined in the literature address the provision of fault tolerance in the structures of these layers, as follows.

The System layer is related to the processing cores and applications. Some works, like [32,33,34,35], sought to carry out the remapping of a task from a defective processor to a healthy unit. The Interface layer provides the communication services that are operated at the NIs. This layer was the focus of investigation in [36,37]. The solutions of these authors included the retransmission of corrupted packets [21,22] and giving support to communication primitives, such as OpenMP and Message Passing Interface (MPI) [38].

The Network layer essentially consists of the routers. Some works address the methods for the protection [19] and verification [22,39] of its internal components. In [40], the authors focused on the switching logic though most implementations involving routing algorithms that bypass defective routers. Many of them include adaptive routing algorithms in 2D [41,42,43,44,45] or 3D topologies [28,31,46]. One way to ensure packet delivery is by using trusted paths [47] or even redundant subnets [38,46]. Multiple path techniques, like flooding, can also be adopted [48].

The Link layer comprises the data links responsible for connecting each router to its neighbors and the processing core. Some studies have employed techniques related to flow control, such as bus encoding [18], retransmission [20], and bandwidth allocation [49]. Others have adopted additional links, such as the duplication of wires and channels [24,50], spare links [41,51], link serialization [26], or by generating new links [25]. The main focus in 3D NoCs is on the TSVs and the problems related to the manufacturing process [27,29]. There are also studies about fault tolerance techniques for unstructured links, such as optical [52] and wireless [53].

#### 2.3.2. Detection and Correction

The basic mechanism for providing fault tolerance in a system is redundancy [54]. The purpose of this mechanism is to detect and, in some cases, to fix errors in the components. Redundancy techniques can be classified as Spatial, Temporal, and Information and are applied in NoCs as follows.

Spatial redundancy involves the addition of circuits, with the replication of modules whose outputs are compared by a voter. It is often performed through the use of Dual Modular Redundancy (DMR) and Triple Modular Redundancy (TMR). When spatial redundancy is implemented in NoCs, it often consists of the replication of links [20,27,28,30,34,38,40,47,50]. The replication of routers [34,35], their internal structures [42,51], the use of adaptive routing tables [55], and the inclusion of checkers and testers in hardware [39] are also classified in this category.

Temporal redundancy consists of the re-execution of an operation resulting in comparison and validation. It is usually implemented by running an algorithm *n* times on the same hardware. In NoCs, some works employ temporal redundancy through the generation of multiple sampling of a message [19,32,48] or through link sections [26].

Information redundancy relies on additional bits for error detection and correction. Error-Detecting Code (EDC) techniques can detect an error incidence, while Error-Correcting Code (ECC) not only detect, but also correct an erroneous data word. Examples include parity [56] and Cyclic Redundancy Check (CRC) [21,36]. Moreover, the replication of the header flit can be adopted to ensure correct routing [23].

#### 2.3.3. Recovery

There are two main types of technique for recovering from an error: Forward Error Recovery (FER) and Backward Error Recovery (BER). FER enables operational continuity in the presence of errors, without having to return to a previous state. Hamming distance, which is a concept widely addressed in the literature, consists of the number of positions in which a current word differs from a previous one. In NoCs, some works use the Hamming code and its variations to correct an error and detect up to two errors in a single data word [19,30,37].

BER techniques ensure the system can return to a previous state when it is considered to be healthy. They use checkpoints or logs and require additional memory elements to preserve these states. In NoCs, BER techniques rely on packet retransmission [20,21,32,49,51]. It is worth noting that FER is more widely used than BER because the latter requires more memory elements, which makes the system more susceptible to SEU faults.

#### 2.3.4. Diagnosis and Repair

Detection and correction are approaches that are sufficient for the operational continuity of a system with transient errors. However, when a system has permanent errors, it is necessary to test and check its components to determine its correctness. Some works offer online test features [21,30,38,39], while others adopt Built-In Self-Test (BIST), a mechanism that enables a system to test itself. In BIST, specific hardware generates stimuli at the inputs of a circuit and compares the output with the correct expected values. Variations of this technique were employed in NoCs [24,26,34,41,45].

After discovering a defective component, it is desirable to disable it and implement a contingency plan, such as reconfiguration, before resuming the execution. In routers, it is possible to employ techniques that can avoid faulty components in the switching logic [21], arbiters [52], and links [26]. However, the focal point of the research is on the reconfiguration of the routing tables, as well as the algorithms needed to bypass faulty routers [28,40,41,42,43,47,55]. The network interfaces are also used for diagnosis and repair [36].

#### 2.3.5. Evaluation and Metrics

Several studies have adopted specific simulators and virtual platforms to evaluate the proposed techniques [26,33,34,39,44,45,49,51,53]. Some of them used dedicated simulators based on the targeted application [25,28,38]. Most of the works carried out a synthesis in Application-Specific Integrated Circuit (ASIC) to obtain a more accurate cost assessment than that provided by the synthesis of programmable logic devices, such as Field Programmable Gate Array (FPGA).

The primary metrics for evaluating fault tolerance techniques in NoCs are area overhead, latency, and power consumption. Other widely used metrics are bandwidth and throughput [26,30,31,33,42,44,45,48,51,56,57]. Some works also estimate the coverage [19,20,24,39,58], the rate [18,29,45,50,53], and the stabilization [55] of faults and errors. Yield [27,34], temperature [28,35], and the number of hops between routers [25,47] are also taken into account when assessing the techniques.

## 3. Router Architecture

In this study, we designed a parameterizable router architecture to evaluate the occupation of resources, propagation of errors, performance, and energy consumption of different combinations of the controllers responsible for data flow regulation, routing, and arbitration. We then implemented these controllers using Moore and Mealy FSMs. This router was partially evaluated in [11], in which we analyzed the different implementations of the routing controllers only.

This article explores the design space for implementing the controllers of an NoC router for finding the best trade-off. It also provides a reference for evaluating the possible combinations of controllers when considering a specific environment or the constraints that are more stringent for the design of a given distributed system. As far as we know, no other work in the literature has investigated the internal mitigation of errors concerning the type of implementation chosen for the router controllers.

A Moore machine defines its output signals according to its current state, whereas the Mealy machine also takes into account its input signals and asserts these outputs during the transition from one state to another (or to the same state).

**Figure 2:**
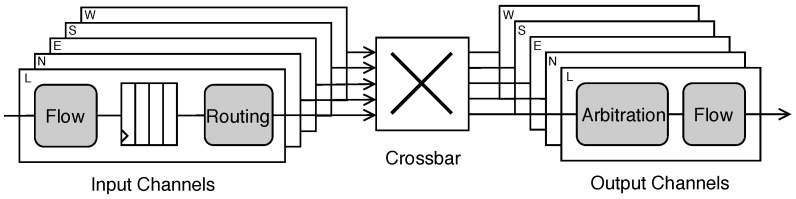
Proposed router architecture.

The architecture of the router was designed with a focus on regularity, flexibility, and low area overhead. To fulfill these requirements, we employed the wormhole switching technique and input buffers capable of storing *n* words. The router had five ports named *Local*, *North*, *East*, *South*, and *West*. The *Local* port was the terminal at which a processing core was attached through a network interface, and the other ports were used to connect the router with its neighbors. Internally, each port was connected to a crossbar that was responsible for the interconnection among the input and output channels that composed the communication ports. Figure 2 shows the architecture of the router.

**Figure 3:**
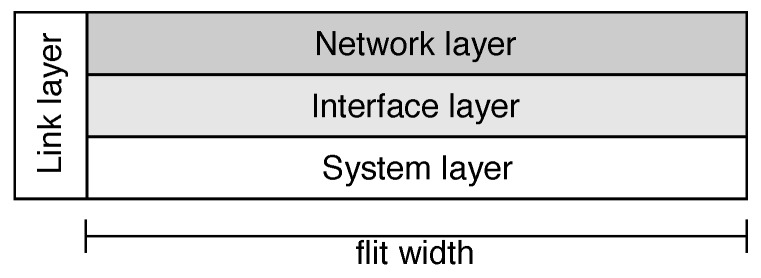
Proposed packet structure [11] (©2019 IEEE).

The novelty of the proposed router architecture was its flexibility to combine different types of FSM in the implementation of the internal controllers. The router was intended to be used in 2D mesh topology networks. Figure 3 shows the proposed packet structure overview.

The general packet format covers all the layers shown in Table 1. The sideband bits were related to the Link layer and comprised the framing tags that defined the begin and the end of the packet. The packet was then structured in one or more mandatory Network flits, optional Interface flits, and the payload flits related to the System layer.

To determine the latency of a flit to traverse the router, it is necessary to assess the number of cycles spent in each sequential logic structure, as presented in Equation (1). As the crossbar was implemented using combinational logic only, this component was not considered in the latency analysis.

(1) $$Latency_{flit}=Cycles_{flow}+Cycles_{buffer}+Cycles_{routing}+Cycles_{arbitration}+Cycles_{flow}$$

In a scenario without router contention, the buffer latency consisted of a single cycle. The internal controllers of the router varied their latency according to its implementation. Each controller required one cycle when using a Moore FSM or none when with a Mealy FSM. Input and output flow controllers must be implemented using the same FSM approach to provide link compatibility. Equation (2) represents the best case latency for a flit to traverse the router.    

(2) $$\begin{array}{cc} Latency_{flit} & =2\times Cycles_{flow}+Cycles_{routing}+Cycles_{arbitration}+1\\ Cycles & =\{\begin{array}{cc} 1 & \mathrm{when}\;\mathrm{Moore}\\ 0 & \mathrm{when}\;\mathrm{Mealy} \end{array} \end{array}$$

Using only Moore-based controllers, a flit needs at least five cycles to traverse a router. In a fully Mealy architecture, only the buffering cycle is required. The following subsections present the architecture of each Moore controller and its Mealy equivalent for performing the same function.

### 3.1. Flow Regulation Controller

The flow regulation controller implemented a four-stage handshake protocol for receiving and sending packets through its input and output channels, respectively. The signals used in these controllers comprised:

- val and ack: flow control signals used for validation and acknowledging the flit transferred through the link.
- wok and wr: write port signals of the input buffer.
- rok and rd: read port signals of the input buffer.

**Figure 4:**
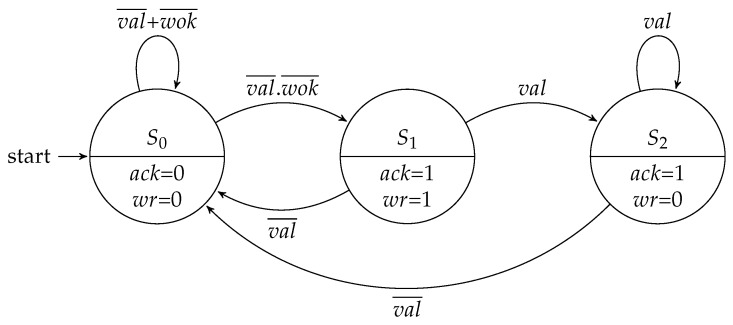
Moore FSM for the input flow regulation controller.

**Figure 5:**
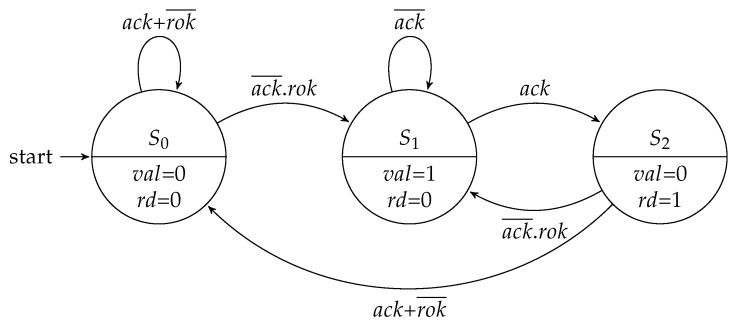
Moore FSM for the output flow regulation controller.

The input and output flow controllers share the same parameter to define the type of FSM to be used. The Moore implementations of these controllers are depicted in Figure 4 and Figure 5.

**Figure 6:**
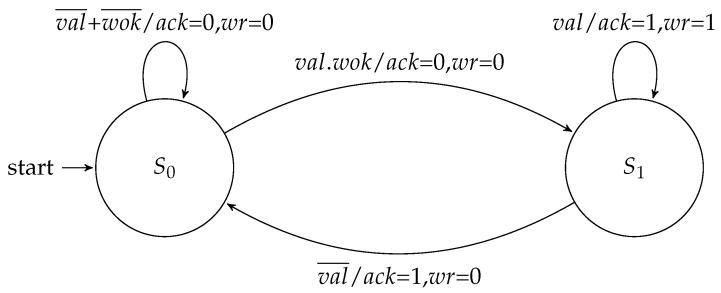
Mealy FSM for the input flow regulation controller.

**Figure 7:**
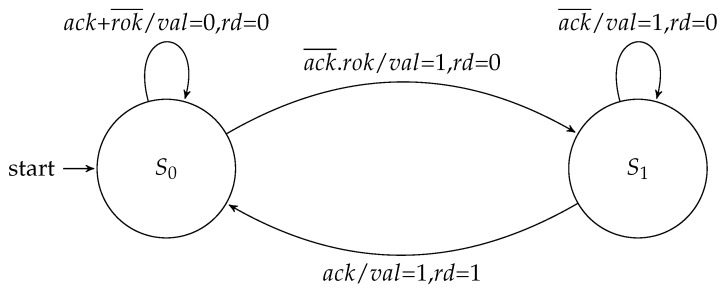
Mealy FSM for the output flow regulation controller.

Figure 6 and Figure 7 show the Mealy variation for the flow regulation controllers. Compared to the Moore machine implementations, each machine saved one state.

### 3.2. Routing Controller

**Figure 8:**
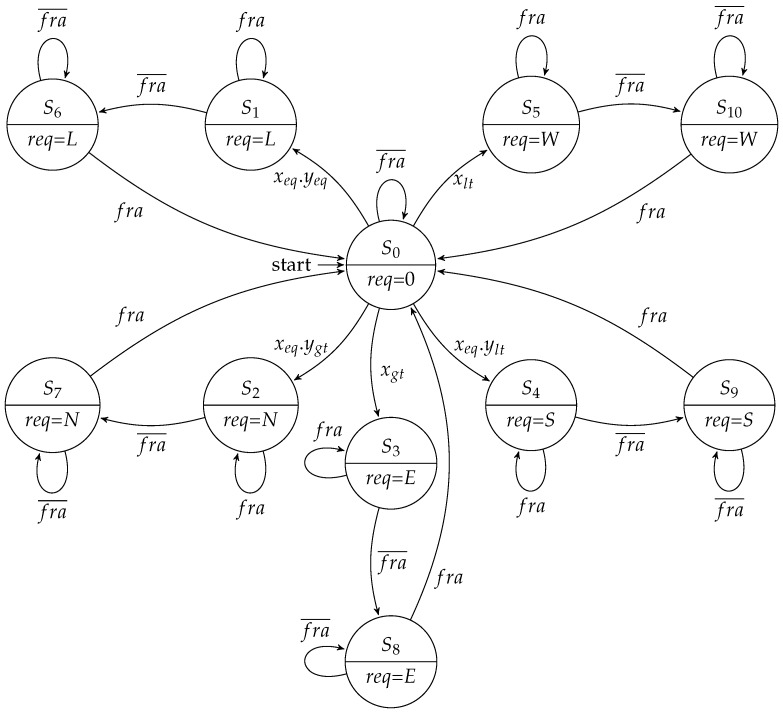
Moore FSM for the routing controller.

The routing controller executed the XY routing algorithm to schedule an output channel. It was composed of a datapath and an FSM. The datapath had comparators that analyzed the destination address enclosed in the packet header. It compared this address with the coordinates of the router to define a set of signals that identify the relationship between these addresses. These signals were named $x_{eq}$, $y_{eq}$, $x_{gt}$, $y_{gt}$, $x_{lt}$, and $y_{lt}$. This XY algorithm started running after the arriving of a tag (fra) that assigned the begin of a packet. The scheduling of an output channel then followed the well known criteria of the XY algorithm: any packet must first travel through the *X* direction, and only when it reaches the same column of the destination node, it can take a path through the *Y* direction. Figure 8 presents the Moore implementation of this controller.

**Figure 9:**
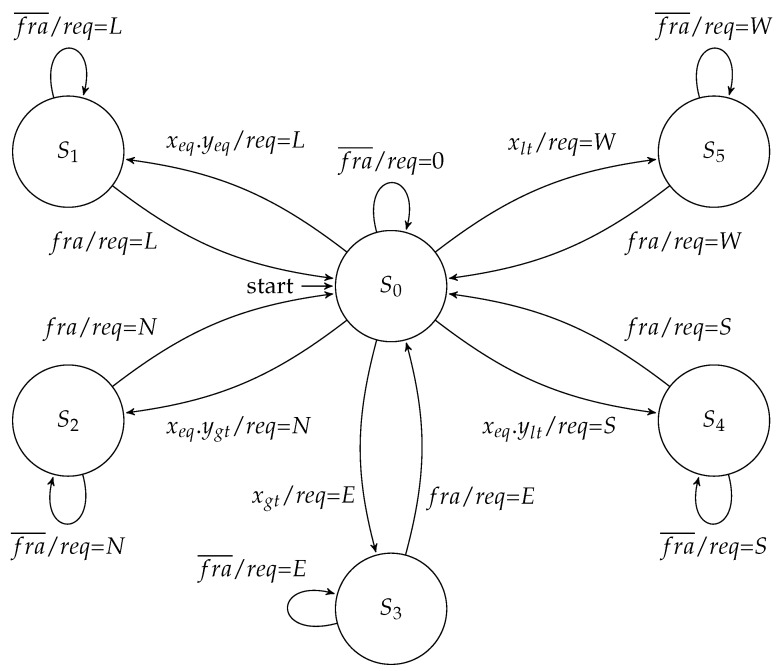
Mealy FSM for the routing controller.

In the Moore approach, when a packet header was received, the machine took a branch to schedule an output channel, and each branch had two states. In the first state, after the packet header was forwarded, the FSM went to the second state, in which it waited for the packet trailer. When this trailer was forwarded, the FSM went back to the idle state (i.e., S0), and the request was de-asserted. On the other hand, in a Mealy implementation (Figure 9), it was possible to save one state for output for each channel scheduling.

### 3.3. Arbitration Controller

**Figure 10:**
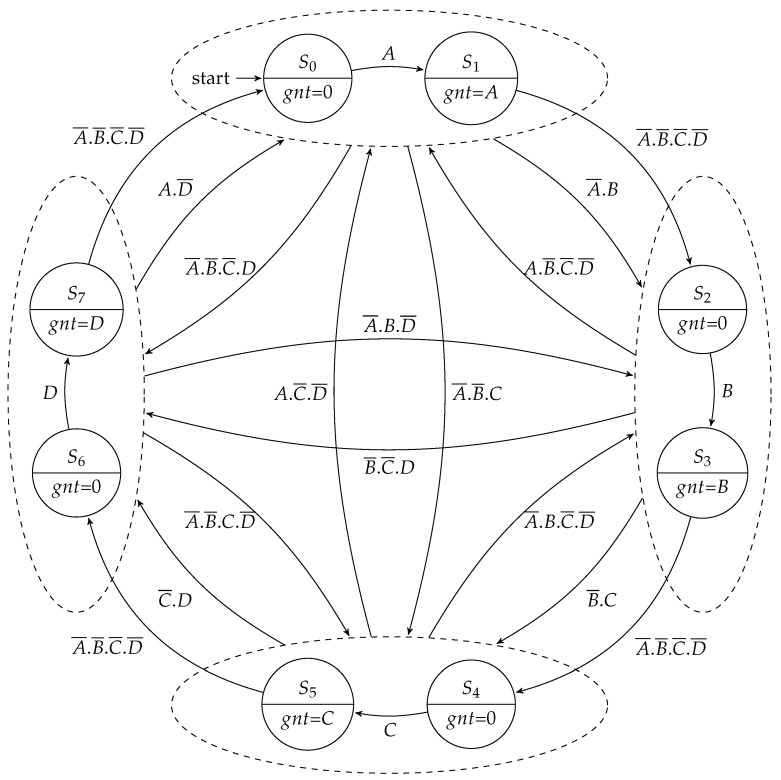
Moore FSM for the arbitration controller.

The arbitration controller consisted of a round-robin arbiter responsible for scheduling the use of the output channel by the packets of the requesting input channels. As there was no provision for loop-back communication in our implementation, each arbiter scheduled up to four requests (namely *A*, *B*, *C*, and *D*), depending on the router address. Similar to the previous controller, it could also be implemented using a Moore or a Mealy FSM. Figure 10 presents a simplified representation of the Moore-based implementation (some transitions are omitted for the sake of clarity).

**Figure 11:**
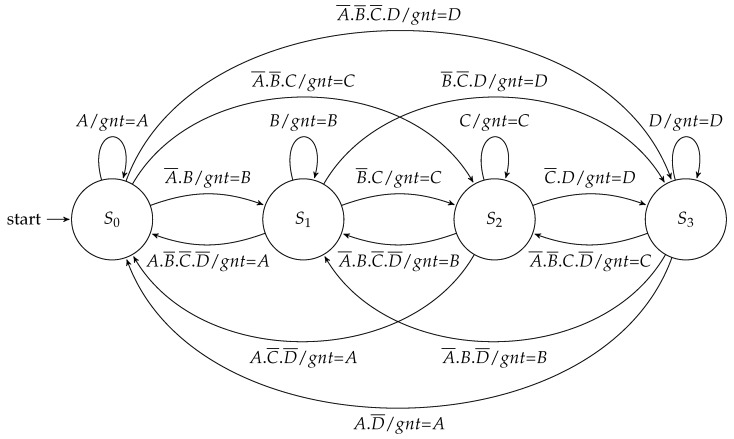
Mealy FSM for the arbitration controller.

As in the design of the routing controller, the Mealy implementation of the arbitration controller (Figure 11) needed only one state for each scheduling branch, and a grant was only given when there existed an active request.

### 3.4. Controller Protection

**Figure 12:**
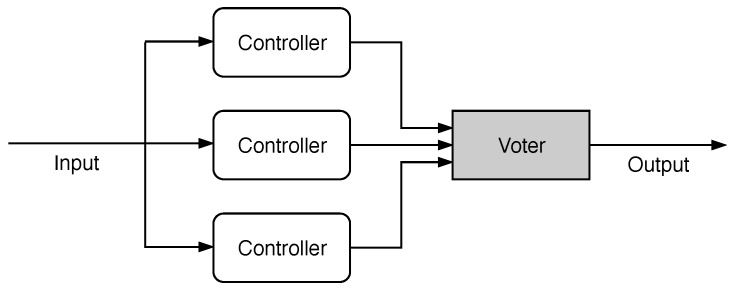
Triple modular redundancy on controllers.

The controllers described above were also implemented in a hardening version. The TMR technique was chosen to protect the FSM of each controller. It consisted of replicating the component in three units, all of them operating over the same input signals. Afterward, the output of each controller is compared by a single major voter, which elected the most common output value, as illustrated in Figure 12.

The TMR technique was selected because it was widely used in reliable systems and due to its ability to mask an error transparently [2]. This technique implied a high resource overhead if applied to complex structures. Meanwhile, in the case of components with few interface signals and few registers, as the focus of this work, it could represent a good trade-off.

## 4. Fault Injection

This section first describes the combinations of the router architecture submitted to verification. Then, it presents the fault injection method and the fault model used for the experiments.

### 4.1. Router Verification

**Figure 13:**
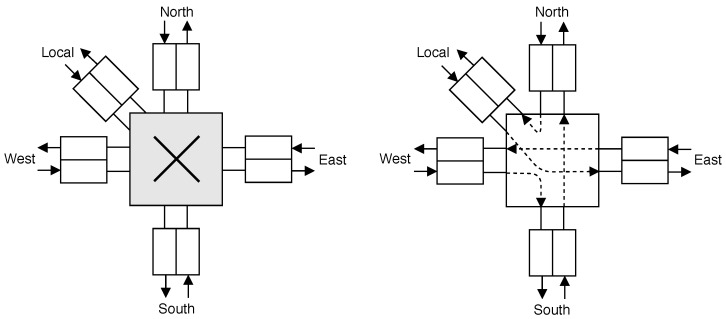
Verification scenario [11] (©2019 IEEE).

In our study, a workload was generated for the evaluation of the router and the fault injection campaign. This workload was designed to inject packets continuously to a fixed set of non-concurrent paths of input and output channels. Thus, it enabled obtaining metrics for the router operating at its highest possible load. The following channel combinations were assigned to comply with the XY routing algorithm requirements: *Local* → *East*; *East* → *West*; *West* → *South*; *South* → *North*; *North* → *Local*. Figure 13 illustrates the connections within the crossbar of the router.

**Figure 14:**
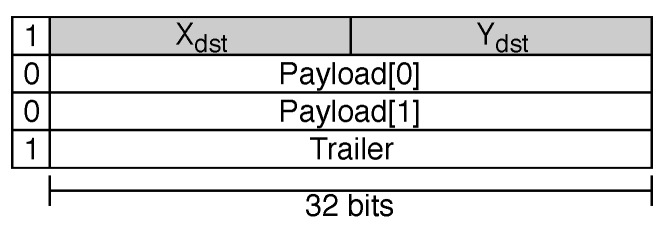
Packet format for verification.

The packet format used for verification is shown in Figure 14. It consisted of a single bit to perform flow control, a single flit as the header, two payload flits, and a trailer. The header flit was used solely to address the coordinates of the destination router. Both the header and the last payload flit (trailer) used “1” as the frame bit, while the regular payload flits used “0”.

In the designed workload, each communication flow comprised the transfer of 4-flit packets, each one composed of a header, a 2-flit payload, and a trailer. Packets of this length are typically used for the transfer of a 128-bit cache line in 32-bit systems.

### 4.2. Fault Injection Environment

There are several different fault injection strategies proposed in the literature. They can be classified into hardware-based injection, software-based injection, simulation-based injection, emulation-based injection, and hybrid injection [59].

The strategy proposed in [60] was adopted for this experiment. The solution was designed originally to inject SEU faults into the registers of a processor and was customized to operate on the proposed router. The technique consisted of a simulation-based fault injection that relied on the use of built-in commands of the ModelSim^®^ simulator. Each iteration of the fault injection strategy included the following stages:

1. Simulating without injection of faults to obtain a golden run.
2. Listing all the registers in the circuit and choosing a random one to inject a fault in it.
3. Randomly determining when the fault will occur within the simulation time.
4. Simulating until the given injection instant.
5. Forcing a bit flip into the selected register.
6. Simulating for the predefined time interval.
7. Comparing the outputs with those from the golden run.

In each experiment, a single fault was injected by inverting the logical value in the target signal. If the output of any external port differed from the golden run, then it was assumed that the fault resulted in an error. For each router configuration, 1000 simulations running for 100 μs were performed. This approach was applied to obtain a more accurate measurement of the error propagation rate in all scenarios. Algorithm 1 presents a pseudo-code that summarizes the steps of the fault injection campaign.

**Algorithm 1** Fault injection campaign.
 1:**set**EndTime = 100 μs 2:**set**TotalRuns = 1000   3:**function**GoldenRun(arch) 4:    simulate(arch) **until** EndTime 5:    **return** arch.outputs 6:
**end function**
   7:**function**FaultInjection(arch, run) 8:    **for** i = 0 to TotalRuns - 1 **do** 9:        flipflop←random(arch.flipflop)10:        simulate(arch) **until** random(EndTime)11:        flipflop←!flipflop12:        simulate(arch) **until** EndTime13:        run(i)←arch.outputs14:    **end for**15:
**end function**
  16:**function**ErrorPropagation(run)17:    **for** i = 0 to TotalRuns - 1 **do**18:        **if** run(i)!=GoldenRun(arch) **then**19:           error←error+120:        **end if**21:    **end for**22:    **return** error23:
**end function**



An architecture that requires more memory elements (i.e., registers) is more susceptible to SEU faults, due to the increased exposure area. For this reason, the number of propagated errors was normalized for a fairer comparison. Equation (3) shows the normalization adopted for the error rate comparisons.

(3) $$Error_{rate}=\frac{|Simulation_{\left(error\right)}|}{|Simulation_{\left(total\right)}|}\times \frac{|Register_{\left(arch\right)}|}{|Register_{\left(reference\right)}|}$$

The number of simulations that propagated an error was divided by the total number of simulations, 1000 in this case. This ratio was then multiplied by the total number of registers in the architecture under simulation by the registers count from a reference architecture, which was given by the most costly one.

## 5. Results

We defined a set of configurations to evaluate the different approaches for implementing the controllers, and each controller was implemented using a Moore or Mealy machine, with or without TMR. For reference, the implementations without TMR were named STD (i.e., Standard). We then synthesized each implementation to obtain its silicon costs, power consumption, and performance metrics. Afterward, we applied the fault injection campaign to measure the error rate. It is worth noting that the router was configured to use handshake flow control, XY routing, round-robin arbiter, flits with 32-bit width, and a 128-bit input buffer.

The architectures were described in VHDL and synthesized using the Intel^®^ Quartus Prime, Version 18.1, targeting the 5CGTFD9E5F35C7 FPGA device of the Cyclone V family. For synthesis, all optimization flags were de-asserted to allow the inference of redundant circuits when applying TMR and also for a more accurate comparison.

The simulations were run using ModelSim-Intel^®^ FPGA Edition. The Quartus Power Analyzer tool was used for power and energy estimation, and it was configured to use a Value Change Dump (VCD) file from the ModelSim simulation as input stimuli.

The different architectures evaluated were identified as STD (Standard), when no fault tolerance technique was applied, and TMR, when the triple modular redundancy technique was used.

All the experiments were conducted on an IBM PC compatible laptop with an Intel i7-4510U processor and 8 GB of RAM, running the Ubuntu Linux 18.04 operating system. We executed 22 syntheses, one per router architecture, and each synthesis lasted about three minutes. The fault injection experiments comprised 1000 simulations per architecture, consuming about six hours to evaluate all the design space considered.

To evaluate the synthesis and resilience results, we employed the metrics most commonly adopted in the literature [54]. Silicon costs are expressed by the number of Look-Up Table (LUTs) and Flip-Flop (FFs) occupied. Performance was given by the maximum operating frequency (F_max_), execution time, and throughput (i.e., the rate of data delivered without errors). Energy costs are given by the total power dissipation and the energy consumed during the time used to deliver the data injected. Finally, the primary metric was the error propagation rate, from which we inferred the fault coverage. The lower the error rate, the higher the fault coverage. The following subsections present the results obtained.

### 5.1. Moore STD versus Moore TMR

Table 2 presents the synthesis results for the experiments that evaluated the Moore-based implementations. As expected, the architecture that did not use redundancy in any of its controllers required the least combinational and sequential elements. Due to a longer critical path, configurations that used redundancy in controllers showed some degradation at the maximum operating frequency. It was observed that the dissipated power practically did not change, even with the presence of a VCD input file. This behavior occurred because an isolated router consumes less than 1% of the resources available on the target FPGA, even in its most expensive configuration. However, these values were useful in obtaining energy consumption considering the simulation time.

The simulation results of the Moore-based configurations are presented in Table 3. Following the fault injection campaign, a minor change in the propagated error rate was observed. This behavior was mainly because the controllers did not account for the largest number of registers. This cost was due to the input buffers.

Considering the maximum operating frequency and given the simulation time, we can obtain the total execution time (t_exe_). This information, combined with the amount of flits transmitted by each of the communication channels, enabled measuring the total router throughput, considering the valid accepted traffic (with no errors). Similarly, by multiplying the total execution time by the power obtained in synthesis, we had the energy consumed by each combination.

In this comparison, the standard version of the router had the highest throughput and the lowest energy consumption. These results indicated that considering the entire router, the slight increase in reliability with protected controllers came at a high price because the performance and energy efficiency were degraded by more than 20%.

### 5.2. Mealy STD versus Mealy TMR

The results obtained from the evaluation of Moore-based controllers showed that the application of redundancy produced a slight reduction in error propagation, but at the price of degrading performance and energy efficiency. Looking for better results, we developed a Mealy variation of each controller.

Table 4 presents the synthesis results for the Mealy-based controllers. As observed in the previous section, the use of TMR led to an increase in the use of combinational and sequential logic and a subsequent reduction in the maximum operating frequency.

As can be seen from Table 5, the application of the hardening technique slightly increased the router reliability, showing a 2% error rate in a fully protected configuration. However, the highest throughput and the highest energy efficiency came from the unprotected version. As Mealy-based controllers had lower latency than their Moore equivalent, this ended up reducing the average latency of packet propagation across the router. This effect occurred because reducing the time a packet stayed on the router also reduced its fault exposure.

### 5.3. Moore versus Mealy

**Figure 15:**
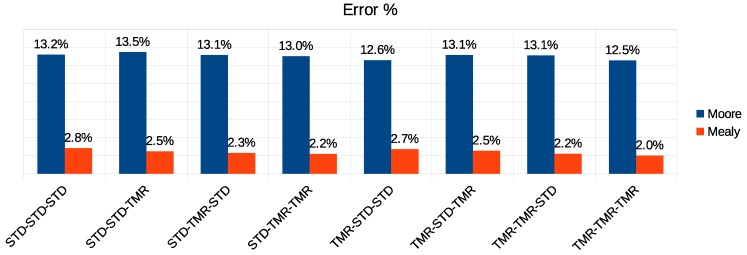
Error propagation rate in the Moore versus Mealy comparison.

**Figure 16:**
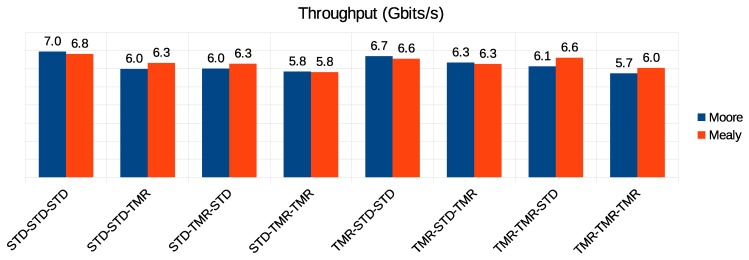
Performance in the Moore versus Mealy comparison.

**Figure 17:**
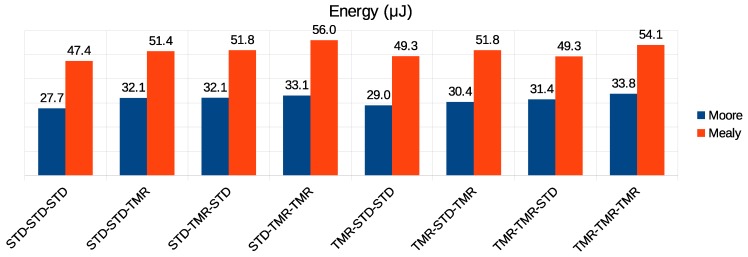
Energy consumption in the Moore versus Mealy comparison.

Figure 15, Figure 16 and Figure 17 present the results of the simulation of all possible combinations of FSM (Moore or Mealy) and implementation (STD or TMR). For instance, the configuration STD-STD-STD defined that both the controllers for flow regulation, routing, and arbitration, respectively, did not implement triple modular redundancy. For this configuration, as Figure 15 shows, the Moore-based controller had an error propagation rate of 13.2%, and the Mealy-based controller had an error rate of 2.8%. It is worth noting that all the controllers of each configuration used the same type of state machine implementation. By analyzing the results shown in this figure, we observed that, for the same type of FSM implementation, the error propagation rate decrease was not significant as the controllers were being protected. However, when comparing the FSM mode, the Mealy-based approach proved to be more resilient, propagating on average 10% fewer errors than the Moore-based implementation for the same combination of controllers.

Figure 16 presents the results regarding the throughput. The results showed almost no variation between the Moore and Mealy implementations of each configuration. However, we noticed that increasing the reliability of the routing and arbitration controllers by using redundancy resulted in throughput degradation. On the other hand, adding TMR to the flow regulation controllers did not degrade communication performance. On the contrary, in some cases, it could result in a slight improvement in throughput.

Figure 17 compares the energy consumption of the different configurations. As we can notice, the Mealy-based configurations consumed approximately 60% more energy than Moore ones in all comparisons. From these results, we can state that the Moore-based implementations were the most energy efficient.

The results presented show that the use of Mealy machines instead of applying TMR was a good alternative to decrease the error propagation rate and improve throughput. However, it must be noted that the Mealy-based controllers were less energy efficient than the ones based on the Moore machine.

### 5.4. Moore and Mealy Combined

With the low effectiveness observed in the application of TMR, along with the high energy consumption inferred from the use of Mealy-based controllers, we looked for solutions that presented a better trade-off. Because the router used in this work was developed focusing on flexibility, having a well defined component interface, it was possible to use mixed Moore and Mealy controllers within the router. Thus, we configured architectures combining the standard (non-protected) versions of Moore and Mealy controllers. Table 6 presents the synthesis results for each one of these combinations.

The increase of LUTs for Mealy-based controllers was expected, due to the additional decoding of the output signals in states. As these machines also needed to encode fewer states, they also presented a decrease in the use of memory elements (i.e., Flip-Flops).

Increasing combinational logic implies a longer critical path of the circuit. As the critical path increased, the maximum operating frequency reduced, which made a fully Moore approach have a maximum operating frequency 71% higher than a configuration using only Mealy controllers, for instance. As explained earlier, the power dissipation remained uniform across all combinations.

Regarding the metrics obtained from simulation (Table 7), we observed a large variation on the error propagation rate. This variation was not only due to the lower use of FFs, but mainly to the retention of data in the input buffers.

**Figure 18:**
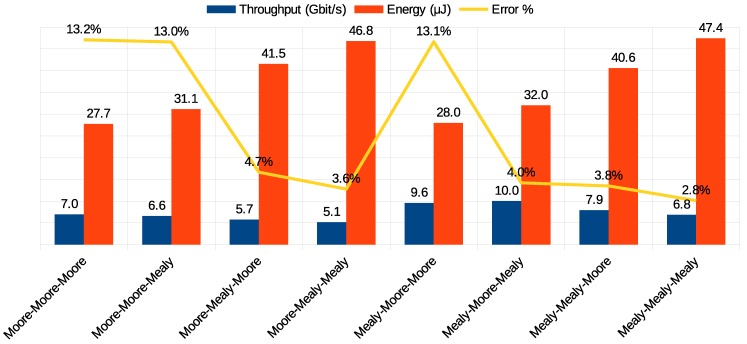
Comparison of Moore and Mealy combined implementations.

A further investigation noted that error propagation was directly related to data retention in buffers. Analysis of previous architectures showed that the speed-up in data forwarding when using Mealy provided increased reliability. Looking at the simulation results of the combined FSM (Figure 18), we can see that there were other combinations besides the fully Mealy one that took advantage of faster data forwarding.

We realized that the throughput was higher when using Mealy machine in the flow regulation controller and Moore machine in the routing controller, and the way arbitration was implemented showed no significant change in performance. The lowest energy consumption was observed when applying Moore on both routing and arbitration controllers.

The application of TMR did not imply a good trade-off in applications with communication or energy restrictions. However, given a router that originally had its controllers implemented through Moore FSMs, the simple migration of the flow regulation controller to Mealy was enough to increase throughput by 37%, with equivalent energy consumption. In reliable systems, the combination that adopted Mealy in flow regulation and arbitration controllers and Moore in routing seemed to be a valid alternative, as its error propagation rate was 4% and energy consumption was only 15% higher in comparison to the baseline version, and in addition, it was the configuration with the highest throughput among all the architectures evaluated.

## 6. Conclusions

Future reliable systems, such as those used in space applications, will incorporate multiple processing cores into an SoC. This processing power is a demand for applications that require high performance in space due to the increasing amount of high resolution sensors and the bandwidth requirements of satellite-ground links. NoCs are the successors of the multi-core interconnection based on shared buses. However, their silicon costs are not negligible, and it is still challenging to provide an architecture that meets the reliability requirements at a low area overhead.

In this context, the main goal of this work was to provide a simplified and parameterizable router architecture and analyze the behavior of its custom architectures under the SEU fault injection. The obtained results showed that the use of Mealy FSMs for the control structures resulted in a significant decrease in the number of propagated errors, at a price of performance degradation and lower energy efficiency. The lower error propagation of the Mealy-based approaches was given mainly due to the occupation rate of the buffers. Each controller that used a Mealy implementation saved a cycle, which sped up data forwarding and caused the packets to be stored for a shorter time.

The article showed which were the best trade-offs that could be achieved in the design of an NoC router, combining the different implementations of its controllers. It also provided a reference for evaluating the possible combinations of controllers when considering a specific environment or the constraints that were more stringent for the design of a given distributed system. As far as we know, no other work in the literature has investigated the inner mitigation of errors concerning the type of implementation chosen for the router controllers.

As future work, we intend to evaluate the proposed router through fault injection in a particle accelerator and then use the router to integrate reliable multi-core systems such as those needed in satellites and other space applications. The source code of the router presented here is available in [61].

## Figures and Tables

**Table 1:** Relationship between the OSI reference model and SoC/NoC layers.

OSI Layers	SoC/NoC Layers	SoC Components
Application	System	Cores
Presentation		
Session	Interface	Network Adapters
Transport		
Network	Network	Routers
Link	Link	Wires
Physical		

**Table 2:** Synthesis results for Moore-based implementations.

Controller			LUTs	FFs	F_max_ (MHz)	Power (mW)
Flow Regulation	Routing	Arbitration				
Moore STD	Moore STD	Moore STD	1 367	750	225.33	625.01
Moore STD	Moore STD	Moore TMR	1 474	780	195.01	625.69
Moore STD	Moore TMR	Moore STD	1 484	790	194.82	625.38
Moore STD	Moore TMR	Moore TMR	1 569	820	189.04	625.26
Moore TMR	Moore STD	Moore STD	1 433	790	215.56	624.75
Moore TMR	Moore STD	Moore TMR	1 539	820	205.51	625.27
Moore TMR	Moore TMR	Moore STD	1 512	830	198.57	624.44
Moore TMR	Moore TMR	Moore TMR	1 621	860	184.88	624.60

**Table 3:** Simulation results for Moore-based implementations.

Controller			Error Rate	t_exe_ (µs)	Throughput (Gbit/s)	Energy (µJ)
Flow Regulation	Routing	Arbitration				
Moore STD	Moore STD	Moore STD	13.2%	44.38	6.95	27.74
Moore STD	Moore STD	Moore TMR	13.5%	51.28	6.00	32.09
Moore STD	Moore TMR	Moore STD	13.1%	51.33	6.01	32.10
Moore STD	Moore TMR	Moore TMR	13.0%	52.90	5.84	33.08
Moore TMR	Moore STD	Moore STD	12.6%	46.39	6.70	28.98
Moore TMR	Moore STD	Moore TMR	13.1%	48.66	6.34	30.43
Moore TMR	Moore TMR	Moore STD	13.1%	50.36	6.13	31.45
Moore TMR	Moore TMR	Moore TMR	12.5%	54.09	5.75	33.78

**Table 4:** Synthesis results for Mealy-based implementations.

Controller			LUTs	FFs	F_max_ (MHz)	Power (mW)
Flow Regulation	Routing	Arbitration				
Mealy STD	Mealy STD	Mealy STD	1374	729	131.77	624.90
Mealy STD	Mealy STD	Mealy TMR	1418	747	121.67	625.78
Mealy STD	Mealy TMR	Mealy STD	1486	760	120.53	624.94
Mealy STD	Mealy TMR	Mealy TMR	1550	780	111.63	625.50
Mealy TMR	Mealy STD	Mealy STD	1389	749	126.50	624.20
Mealy TMR	Mealy STD	Mealy TMR	1454	767	120.61	625.29
Mealy TMR	Mealy TMR	Mealy STD	1524	780	126.92	625.16
Mealy TMR	Mealy TMR	Mealy TMR	1586	800	115.69	625.33

**Table 5:** Simulation results for Mealy-based implementations.

Controller			Error Rate	t_exe_ (µs)	Throughput (Gbit/s)	Energy (µJ)
Flow Regulation	Routing	Arbitration				
Mealy STD	Mealy STD	Mealy STD	2.8%	75.89	6.83	47.42
Mealy STD	Mealy STD	Mealy TMR	2.5%	82.19	6.33	51.43
Mealy STD	Mealy TMR	Mealy STD	2.3%	82.97	6.28	51.85
Mealy STD	Mealy TMR	Mealy TMR	2.2%	89.58	5.82	56.03
Mealy TMR	Mealy STD	Mealy STD	2.7%	79.05	6.56	49.34
Mealy TMR	Mealy STD	Mealy TMR	2.5%	82.91	6.27	51.84
Mealy TMR	Mealy TMR	Mealy STD	2.2%	78.79	6.62	49.26
Mealy TMR	Mealy TMR	Mealy TMR	2.0%	86.44	6.04	54.05

**Table 6:** Synthesis results for Moore and Mealy combined implementations.

Controller			LUTs	FFs	F_max_ (MHz)	Power (mW)
Flow Regulation	Routing	Arbitration				
Moore	Moore	Moore	1367	750	225.33	625.01
Moore	Moore	Mealy	1353	744	200.64	624.50
Moore	Mealy	Moore	1412	745	150.53	625.14
Moore	Mealy	Mealy	1370	739	133.53	624.61
Mealy	Moore	Moore	1364	740	223.71	625.86
Mealy	Moore	Mealy	1356	734	195.50	625.72
Mealy	Mealy	Moore	1420	735	153.99	624.95
Mealy	Mealy	Mealy	1374	729	131.77	624.90

**Table 7:** Simulation results for Moore and Mealy combined implementations.

Controller			Error Rate	t_exe_ (µs)	Throughput (Gbit/s)	Energy (µJ)
Flow Regulation	Routing	Arbitration				
Moore	Moore	Moore	13.2%	44.38	6.95	27.74
Moore	Moore	Mealy	13.0%	49.84	6.57	31.13
Moore	Mealy	Moore	4.7%	66.43	5.74	41.53
Moore	Mealy	Mealy	3.6%	74.89	5.15	46.78
Mealy	Moore	Moore	13.1%	44.70	9.57	27.98
Mealy	Moore	Mealy	4.0%	51.15	10.01	32.01
Mealy	Mealy	Moore	3.8%	64.94	7.90	40.58
Mealy	Mealy	Mealy	2.8%	75.89	6.83	47.42
